# Thermal Desorption Analysis of Hydrogen in Non-hydrogen-Charged Rolling Contact Fatigue-Tested 100Cr6 Steel

**DOI:** 10.1007/s11249-017-0947-0

**Published:** 2017-11-25

**Authors:** A. D. Richardson, M.-H. Evans, L. Wang, R. J. K. Wood, M. Ingram

**Affiliations:** 1 nCATS, Faculty of Engineering and the Environment, University of Southampton, Southampton, UK; 2 Afton Chemical Corporation, Bracknell, UK

**Keywords:** Thermal desorption analysis, Hydrogen diffusion, Rolling contact fatigue, White etching cracks, Rolling element bearings, Wind turbine gearbox bearings

## Abstract

Hydrogen diffusion during rolling contact fatigue (RCF) is considered a potential root cause or accelerator of white etching cracks (WECs) in wind turbine gearbox bearing steels. Hydrogen entry into the bearing steel during operation is thought to occur either through the contact surface itself or through cracks that breach the contact surface, in both cases by the decomposition of lubricant through catalytic reactions and/or tribochemical reactions of water. Thermal desorption analysis (TDA) using two experimental set-ups has been used to measure the hydrogen concentration in non-hydrogen-charged bearings over increasing RCF test durations for the first time. TDA on both instruments revealed that hydrogen diffused into the rolling elements, increasing concentrations being measured for longer test durations, with numerous WECs having formed. On the other hand, across all test durations, negligible concentrations of hydrogen were measured in the raceways, and correspondingly no WECs formed. Evidence for a relationship between hydrogen concentration and either the formation or the acceleration of WECs is shown in the rollers, as WECs increased in number and severity with increasing test duration. It is assumed that hydrogen diffusion occurred at wear-induced nascent surfaces or areas of heterogeneous/patchy tribofilm, since most WECs did not breach the contact surface, and those that did only had very small crack volumes for entry of lubricant to have occurred.

## Introduction

Driving mechanisms for white structure flaking (WSF) and white etching cracks (WEC) in wind turbine gearbox bearings are highly contested. One of these suggested drivers is hydrogen, where hydrogen that is either sourced from the lubricant or water contamination is released during operation and diffuses into the bearing steel, diffusion of hydrogen leading to an ‘embrittlement’ of the steel and promoting WEC formations [1–7].

During rolling contact, hydrogen formation and diffusion into the steel could occur by a number of mechanisms,

Through surface cracks where water or lubricant entry allows for a local release of hydrogen ions by tribochemical reactions at nascent crack tips [8].
Through wear-induced nascent surfaces in areas of high slippage or heterogeneous tribofilm, hydrogen being generated by the decomposition of lubricants through catalytic reactions [9, 10] and tribochemical reactions of water.
(1) Chemical reaction causing formation of hydrogen cations, which get adsorbed on the cathodic steel surface before diffusing into the steel. The deposition rate of hydrogen being enhanced in the case of high electric field strength and thus high ion mobility or due to increased wear; (2) atomic hydrogen formation through thermal dissociation as a result of discharge in the lubrication gap [7].

In all cases, it is the highly mobile mono-atomic form of hydrogen (H) that diffuses through the material matrix becoming potentially trapped within the steel. The mechanism of hydrogen diffusion through wear-induced nascent surfaces is supported by researchers who have found hydrogen to have diffused during RCF testing [5, 9, 11–13] with measured concentrations of between 0.1 and 4.2 ppm [5, 9, 11], the concentration of hydrogen measured also being proportional to the wear on the steel during sliding tests [9].

Hydrogen can remain in mono-atomic (H) form as an interstitial solute retaining its mobility in high strength steels [11]. After diffusion, hydrogen can exist as either

1. ‘Non-mobile’, non-diffusible hydrogen that is strongly trapped or residual at irreversible sites.
2. ‘Mobile’ or ‘diffusible’ hydrogen that is weakly trapped at reversible sites [12–15].

Traps can include inclusions, cracks, grain boundaries, carbides, microvoids, retained austenite and areas of local plastic deformation where the density of crystal defects (dislocations) is large and hydrogen binding with these crystal defects occurs [14, 16–26]. Traps can be split in terms of their desorption temperature and binding energies. Traps with low binding energies and desorption temperatures are referred to as weak reversible traps, strong binding energies and higher desorption temperatures referred to as strong irreversible traps. Traps can also be sub-categorised as either physical or attractive (or a combination of the two). Physical traps are those where hydrogen is trapped by the way of mobile hydrogen’s random walk, and attractive traps are those such as electrical fields (e.g. electronegative impurity atoms) and temperature/stress gradients [27].

It is considered that strongly trapped hydrogen or hydrogen in its molecular form is not harmful regarding WSF, it being the diffusible hydrogen that ‘embrittles’ the steel; however, recombination of hydrogen to molecular gas can cause hydrogen-induced cracking in steels. It has also been demonstrated that small cracks within the steel matrix can act as strong hydrogen traps rendering the hydrogen non-threatening and unable to assist in the mechanisms of hydrogen embrittlement [26]. It is, however, noted that trapping and saturation of cracks with hydrogen could eventually lead to an internal pressure buildup stimulating crack growth. A counter-argument to this is that saturation of the crack could alleviate the ability for crack face rubbing/beating to occur during RCF [28], where crack rubbing/beating is proposed to generate WEAs [28, 29]. It is suggested that in the formation process of WEAs, hydrogen serves only to enhance brittle cracking, forcing cracks open and inducing additional crack rubbing/beating [28].

There are a number of theories explaining the detrimental effect of hydrogen on steel; however, there is no agreement as to which theory explains the effect of hydrogen on bearing steels. Popular mechanisms include hydrogen-enhanced localised plasticity (HELP), hydrogen enhancing the mobility of dislocations ahead of the crack tip resulting in localised plasticity, hydrogen-enhanced decohesion (HEDE), hydrogen increasing lattice strength by a reduction in cohesive binding energy lowering the stress required for cleavage and hydrogen-enhanced strain induces vacancy (HESIV), hydrogen diffusion towards vacancies and nanovoids, their formation being assisted under strain leading to ductile crack growth by localised slip [12, 30, 31].

To measure the concentration of diffusible hydrogen in bearing steels, a number of techniques are typically used: the mercury method [32], thermal desorption analysis (TDA) using thermal conductivity detection (TCD) (e.g. gas chromatography (GC) or hot carrier gas extraction [32]), TDA coupled with a mass spectrometer (TDMS) [10, 14, 29, 33, 34] and secondary ion mass spectrometry (SIMS), used to measure the local concentration of hydrogen in specimens and at defects such as inclusions [35, 36]. The primary method for measuring hydrogen in weld steels under the BS ISO 3690 [32] has been the mercury method; however, due to the health hazards when using mercury and the long analysis times involved other methods for measuring hydrogen have been exploited. TDA is one of the more popular methods, where negligible differences in measurable hydrogen concentrations between TDA methods and the primary mercury methods have been found [37, 38], and thus TDA methods are consequently recognised in the BS ISO 3690 [32] for measuring hydrogen in steel welds. Consequently, TDA has been used to measure the concentration of diffusible hydrogen in bearing steels in a number of studies [10, 17, 28, 29, 33, 39–42].

TDA using a TCD can be classified into two methods,

1. Hot carrier gas extraction where the sample is heated at high temperature (up to 400 °C), the diffusible hydrogen desorbed out being measured continuously, where TDA can also be carried out by ramping the temperature gradually at a pre-defined rate (°C/min).
2. Where a sample is placed into a chamber that is heated at relatively low temperature (typically between 45 and 150 °C), hydrogen being measured separately after collection [32].

Potential downfalls to elevated temperature analysis using the hot extraction method lie in the reduction in analysis time and thus a potential increase in the rate of hydrogen desorption from the specimen, resulting in a greater release of hydrogen than that which might desorb out under ambient conditions [37]. The measured concentrations obtained at higher temperatures are a true representative concentration of the hydrogen measured. Other factors that may influence the TDA result include the effect of oxide layers at the surface. It has been observed that hydrogen trapped in the steel reacts with oxygen at the surface during desorption to form water molecules, producing additional unwanted peaks in the TDA spectra [37, 43].

There is debate between standards and studies employing TDA as to what is defined as diffusible or non-diffusible hydrogen. At ≤ 400 °C, desorbed hydrogen is defined by BS ISO 3690 standards [32] as diffusible hydrogen, elevated temperatures > 400 °C being used to measure strongly trapped residual hydrogen, and this is in contrast to the Australian standard for example that defines significant amounts of residual hydrogen are released > 150 °C [44]. TDA conducted on bearing steels, as discussed above, however, does show deposition peak ≤ 400 °C corresponding to diffusible hydrogen, the desorption peak occurring at elevated temperatures > 400 °C corresponding to non-diffusible strongly/residually trapped hydrogen. Since a large number of parameters can affect TDA results, strict measures must be taken. The important parameters to consider are,

1. Heating method (rate, duration, upper temperature).
2. Weight of samples (affects lower detection limit of hydrogen).
3. Geometry of specimens (surface area of samples during TDA).
4. Time to start of TDA (length of time samples are at temperature before TDA or before freezing), where freezing of the sample is used to ensure that as much diffused hydrogen remains trapped before conducting TDA.
5. Freezing method [liquid nitrogen (LiqN)] or dry ice; the colder the temperature, the higher the hydrogen’s mobility and thus effusion is inhibited.
6. Specimen surface preparation (removal of contaminants such as moisture, oxide layers and lubricant residue).
7. Precision of TDA instruments (resolution, calibration and minimum hydrogen detection limits).

To investigate the role of hydrogen diffusion in WTGBs, this study uses TDA to measure the diffusible hydrogen concentration in FAG-FE8-tested cylindrical roller thrust bearings, this being a continuation of the works conducted previously by the authors [45, 46]. Many previous investigations have concentrated on the analysis of pre-hydrogen-charged RCF test specimens [4, 6, 29, 33, 39, 41, 47–52], where it is generally noted that pre-charging decreases RCF life in bearing steels that fail from WSF. This study uses TDA to measure the concentration of diffusible hydrogen in non-hydrogen-charged specimens to better simulate the environments experienced in service. Finally, the link between the role of hydrogen and the propensity for WEC formations has been explored through the combination of TDA and extensive metallographic analysis conducted in a parallel paper by the authors of this manuscript [ref paper 1].

## Materials, Techniques and Experimental Methods

### Rolling Contact Fatigue Testing

RCF testing was conducted on two 100Cr6 steel cylindrical roller thrust bearings (CRTBs) on an FAG-FE8 test rig under non-hydrogen-charged test conditions. Each bearing has 15 individual rollers mounted in a brass cage sandwiched between two washer raceways. The test rig, set-up and conditions are more extensively detailed in [ref paper 1], where metallographic analysis has been conducted on the same test bearing rollers and raceways analysed in this study. The test conditions are shown in Table 1.

**Table 1:** FAG-FE8 RCF test conditions

*Test system*	
Test rig	FAG-FE8
Test sample	Cylindrical roller thrust bearings
Bearing type	F-562831-01/81212
Bearing dimensions roller/raceway	15× rollers, diameter = 11 mm, length = 11 mm. 2× raceway, outer diameter = 95 mm, inner diameter = 60 mm, thickness of each raceway = 7.5 mm. Thickness of compiled bearing rollers + brass cage and raceways = 26 mm
*Oil properties*	
Oil type	Automotive gear oil, fully formulated semi-synthetic (ISO VG64)
Viscosity	64 cSt (40 °C), 9.5 cSt (100 °C)
Pressure viscosity coefficient (*α*)	6.6 GPa^−1^
Dynamic viscosity *η*_o_ (100 °C)	0.0046 Pas
Oil additives	Sodium and calcium anti-corrosion sulphonates, ZDDP anti-wear, VI improvers and friction modifiers
*Bearing material properties*	
Washer/roller/cage material	Martensitic 100Cr6 steel/martensitic 100Cr6 steel/brass
Hardness roller/washer	765/590 HV
Surface roughness (*R*_q_) roller/washer	0.09/0.50 μm
*Test conditions*	
Rotational shaft speed	750 rpm
Axial load	60 kN
Max contact pressure	~1.5–1.9 GPa (depending on contact length used between 7 and 9 mm, 9 mm used in this study)
Bearing/oil temperature	100 °C
Minimum film thickness (*h*_min_)	0.053 μm
Lambda ratio	0.1
Slide to roll ratio (SRR)	± 12.5% rising to the edges of the raceway, pure rolling exists at the centre of bearing contact
*Test durations*	
Test number 1/2/3/4/5/6/7/8	0/2/4/6/6-repeat/12/16.5/18 (hours duration)
*Subsurface shear stresses*	
Max orthogonal shear stress (*τ*_o, max_)	~ 375–475 MPa (acting @ a depth below the contact surface of ~ 92 μm)
Max unidirectional shear stress (*τ*_uni, max_)	~ 456–578 MPa (acting @ a depth below the contact surface of ~ 145 μm)

Minimum oil film thickness (*h*_min_) between rollers and washer raceways is calculated using the Hamrock and Dowson visco-elastic equation [53, 54], see Eq. (1). Lambda ratio (*λ*) has been calculated based upon *h*_min_, and the roughness (*R*_q_) values are given in Table 1, the bearing running in boundary lubrication throughout RCF testing; see Eq. (2).

### Thermal Desorption Analysis (TDA)

**Fig. 1:**
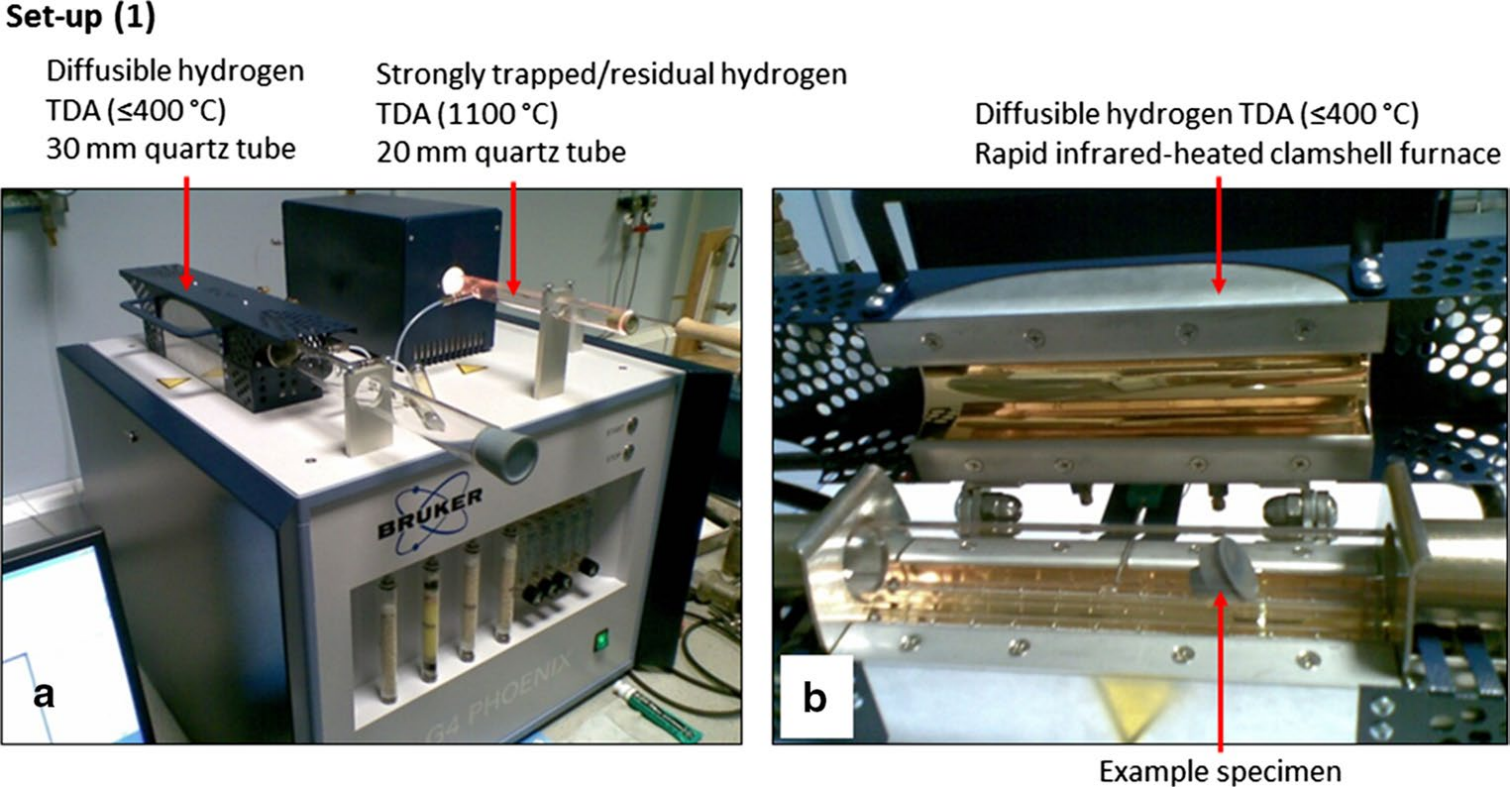
TDA equipment set-up (1). **a** Diffusible and strongly trapped/residual hydrogen analysis tubes and **b** infrared-heated clamshell furnace. Adapted from [55]

To detect any hydrogen that may have diffused and become trapped in the steel during RCF testing, TDA was performed using two different experimental set-ups under BS ISO 3690 standards [32] as detailed below. See Fig. 1 for the respective TDA equipment set-ups.

1. TDA at ≤ 400 °C (diffusible hydrogen) and 1100 °C (strongly trapped/residual hydrogen) was performed on a Bruker G4 Phoenix DH utilising the carrier gas (nitrogen) hot extraction method with TCD of the desorbed hydrogen. A rapid infrared-heated clamshell furnace with a 30-mm-diameter quartz specimen chamber heats up the sample material. For TDA at 1100 °C, a 20-mm-diameter wire-heated tube is equipped. The system uses the hot extraction method, and the system is heated up to 400 or 1100 °C before the sample is placed into the chamber, the sample only being placed once temperature is reached. It is expected that the sample temperature rapidly heats until 400 °C is reached throughout including the sample core. The carrier gas transports the desorbed gases through molecular sieve and Schuetze reagent, and the Schuetze reagent converts CO, CO_2_, H_2_O together with other interfering substances and is removed from the carrier gas by the molecular sieve before the TCD so that only hydrogen is measured. The G4 phoenix is an open system, and thus at the inlet of the analysing tube more carrier gas is being supplied than is being released from the outlet preventing the entry of ambient air into the system. The minimum concentration of hydrogen measurable by the analyser is 0.05 ppm (depending upon sample weight), and the precision is ± 0.05 ppm. Hydrogen detection levels during measurement indicated that all hydrogen was removed within the sensitivity of the instrument within 20 min, and therefore the measurement time was set specifically to 20 min for all tests.
2. Ramped TDA was conducted using GC method at a rate of 25 °C/h up from room temperature to a final temperature of 300 °C (diffusible hydrogen) using an in-house TDA system. TDA using this set-up was only conducted on the 0-h control, 6-h and 18-h RCF-tested bearings. The sample is heated at a linear rate using a resistance tube furnace, a thermocouple being used in close proximity to the sample to measure the temperature. The GC is calibrated with a helium gas containing 60.7 ppmv (part per million per volume) hydrogen before measurement. Helium is used as the carrier gas and flows at a constant rate of 10 ml/min. The carrier gas transports the desorbed gases from the sample through the GC system. The mix of gases is injected into the GC column every 3 min, where the mix of gases is separated and detected by a pulsed discharge ionisation detector. The hydrogen desorption rate (ppm/min) is then measured. Analysis time was conducted over 1250 min, the final temperature of 300 °C being reached after 615 min.

#### Sample Preparation

Prior to TDA, necessary sample preparations post-RCF testing are required to minimise losses and control contamination in the hydrogen detection process.

Once testing had finished, the bearing was removed, cleaned of residual oil and immersed in LiqN under BS ISO 3690 standards [32] prior to TDA. The downtime between the end of RCF tests and immersion in LiqN was kept to a minimum where possible to mitigate any potential losses of hydrogen. The immersion in LiqN ensures that as much mobile diffusible hydrogen remains trapped in the steel bearing before TDA. LiqN at − 196 °C inhibits hydrogen from effusing out of the steel bearing; however, some hydrogen will still desorb out of the steel overtime.

**Fig. 2:**
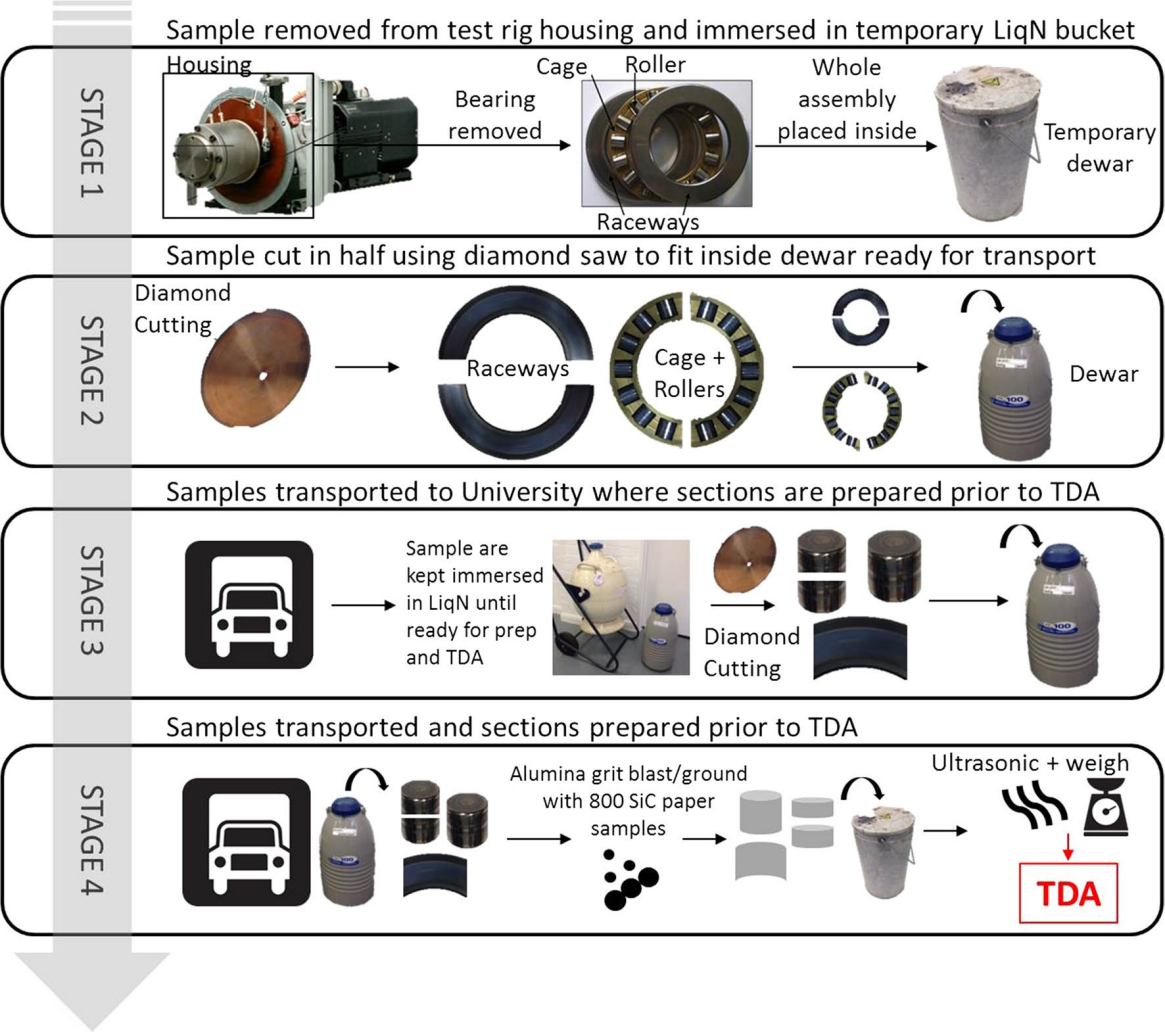
Flow diagram demonstrating the stages of sample preparation post-RCF testing and before conducting TDA

Prior to TDA, the bearing was prepared into the relevant samples to be analysed. The raceway washers were cut into ~ 20 × 20 mm square sections and the rollers kept whole or cut into halves to investigate the influence of outer and inner roller zones. Cutting time was kept to a minimum to limit the desorption of hydrogen (< 1 min). In set-up 1, single and multiple rollers and raceway washer sections were analysed, only single roller and raceway sections being analysed in set-up 2. The rollers analysed were either non-spalled or spalled to investigate the influence of spall sites as exposed surfaces/cracks for additional hydrogen entry into the bearing steel during operation. The samples were kept continuously immersed in LiqN for a period of 2–15 days under BS ISO 3690 standards [32] until TDA was conducted. Before TDA samples were lightly blasted with alumina grit for < 60 s to remove contaminants, residues and the oxide layer, this is with the exception of the 0-h (control) roller and raceway analysed by set-up 2 which were lightly ground using 800 grit SiC paper. The samples were then ultrasonically cleaned in acetone for 60 s to remove surface contaminants, air-dried and then weighed on a mass balance before being placed in the TDA analyser tube. A flow diagram of the sample preparation process prior to TDA analysis is illustrated in Fig. 2.

## Results

### Thermal Desorption Analysis

**Fig. 3:**
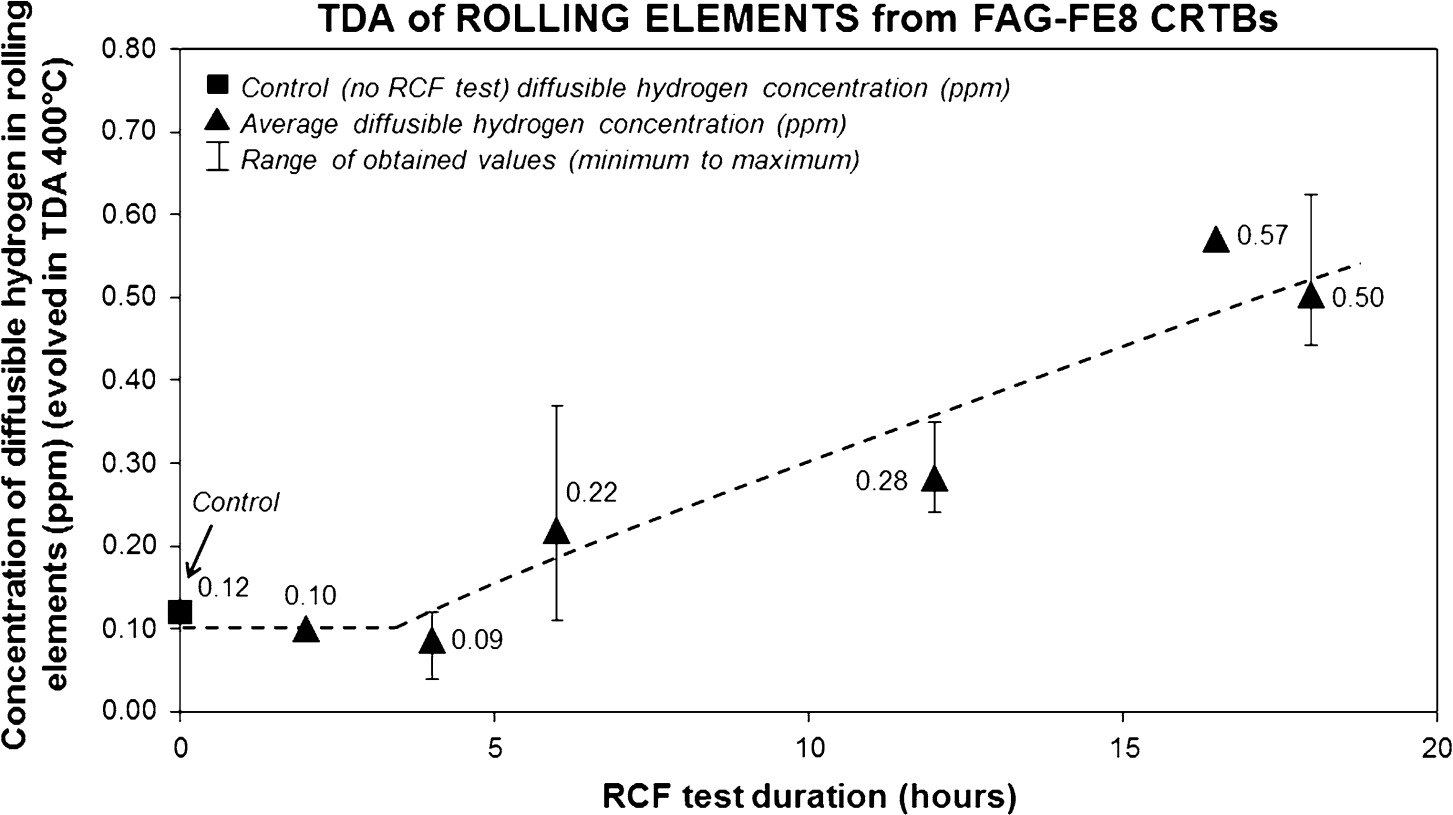
Average concentrations of diffusible hydrogen measured by TDA at ≤ 400 °C using set-up #1 in the rolling elements post-RCF testing. See Table 2 for individual hydrogen TDA measurements

**Fig. 4:**
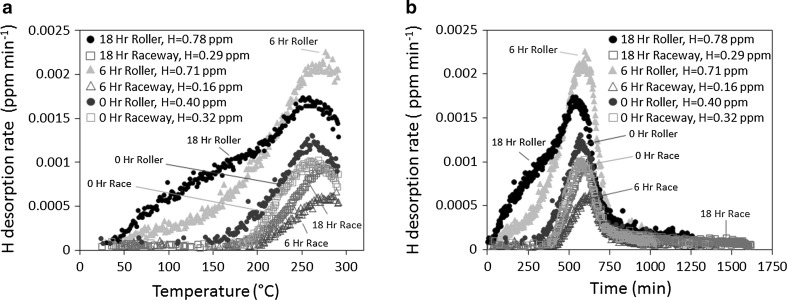
Hydrogen desorption curves for 0-, 6- and 18-h RCF-tested roller and raceway washer section analysed by ramped TDA to 300 °C using set-up 2 including the total diffusible hydrogen concentration measured. **a** Hydrogen desorption rate (ppm/min) versus desorption temperature (°C). **b** Hydrogen desorption rate (ppm/min) versus analysis time (min). See Table 2 for individual TDA measurements

The results from the TDA are detailed below and displayed in Table 2 and Figs. 3 and 4. Results have shown,

**Table 2:** Concentration of diffusible hydrogen (ppm) measured by TDA using set-ups 1 and 2

Bearing section	0 h (ctrl)		2 h	4 h	6 h		12 h	16.5 h	18 h	
*Set-up 1 (TDA @ 400* *°C), Set-up 2 (TDA to 300* *°C @ 25* *°C/hr)*										
Rolling element	SU 1	SU 2	SU 1	SU 1	SU 1	SU 2	SU 1	SU 1	SU 1	SU 2
1× element		0.40		0.04, 0.10, 0.10, 0.12	0.11, 0.13	0.71	0.24, 0.27		0.46, 0.45, 0.35^c^	0.78
2× elements	0.12		0.10		0.32, 0.21, 0.18, 0.37^a^		0.28, 0.35			
3× elements								0.57^b^		
2× inner halves 2× outer halves									0.57, 0.44 0.63, 0.58	
Raceway										
1× section	0.07	0.32		0.07	0.02	0.16	0.014	0.01	0.12	0.29
2× sections									0.15	
*TDA @ 1100* *°C*										
Rolling element										
3× element						0.30^b,d^				
Raceway										
1× section		0.10								

All samples with non-spalled contact surfaces unless otherwise stated

SU 1 or SU 2 denotes set-up 1 or set-up 2

^a^TDA results from 6-h repeat RCF test

^b^1× spalled roller, 1× non-spalled roller, 2× non-spalled roller ‘halves’ analysed simultaneously

^c^The rolling element analysed was spalled on the contact surface

^d^Analysed post-1st round of TDA at 400 °C. Oxide layer was removed with a file and wire brush prior to TDA and was re-weighed

- Negligible (close to the detection limit of the instrument) concentrations of diffusible hydrogen were measured in the raceway sections across 0–18 h (0.01–0.15 ppm) using TDA set-up 1 at 400 °C, these being similar to the 0-h sample (0.02–0.12 ppm).
- Elevated concentrations of 0.29, 0.16 and 0.32 ppm were measured in the raceway sections at 18, 6 and 0 h, respectively, using TDA set-up 2.
- For TDA of a 2-h raceway section at 1100 °C using set-up 1, the evolved hydrogen curve showed a double hump. The first hump, relating to diffusible hydrogen (≤ 400 °C) measured a negligible concentration of ~ 0.01 ppm, and the second hump relating to strongly trapped/residual hydrogen also measured a negligible concentration of 0.10 ppm.
- Elevated concentrations of diffusible hydrogen were measured in the rollers. Increased concentrations have been measured for longer test duration across 0–18 h using TDA set-up 1. TDA using set-up 2 showed elevated diffusible hydrogen concentrations of 0.78, 0.71 and 0.40 ppm for the 18, 6 and 0 h rollers, respectively.
- TDA at 1100 °C using set-up 1 was conducted post-1st round TDA at 400 °C on the same 3 × 16.5 h rollers. A concentration of 0.30 ppm was measured, representing the strongly trapped/residual hydrogen.
- Through TDA of outer and inner roller halves at 18 h, no significant differences between hydrogen concentrations were found.
- No considerable differences were found between spalled and non-spalled rollers.

From the TDA curve in Fig. 4a, it can be seen that the 18-h roller shows a shallow desorption hump between 0 and ~ 50–75 °C, with steady desorption between ~ 50 and 200 °C. Another more pronounced desorption peak is observed just beyond at ~ 200–300 °C. For the 18-, 6- and 0-h raceway sections, a single desorption peak is observed just beyond 175 °C. A single desorption peak is observed just beyond ~ 150 °C for the 0-h (control) roller. The 6-h roller shows a steady desorption between ~ 0 and 150 °C; beyond this a desorption peak is observed at ~ 200–300 °C. The desorption curves presented in Fig. 4 show that hydrogen started to desorb out right away for the 18- and 6-h rollers and at just after ~ 250–375 min for the 18-, 6-, and 0-h raceway sections and the 0-h roller after TDA had started. TDA indicates that all diffusible hydrogen was fully desorbed by the time the analysis had finished at 1750 min.

## Discussion

### RCF Testing

This study has used non-hydrogen charged testing to better simulate the conditions experienced in service by wind turbine gearbox bearings, where many previous investigations have used pre-charging to accelerate WEC formations. The FAG-FE8 test rig, however, have differing dynamics to those experienced by wind turbine gearbox bearings during service. On the other hand, one similarity between the FAG-FE8 and those experienced in service is slip, this being discussed in further detail in [ref paper 1]. Slip between roller and raceway can cause metal-to-metal contact, resulting in the exposure of wear-induced nascent surfaces [9, 10]. It has been demonstrated that hydrogen diffusion is increased in the presence of slip, where hydrogen is generated as a result of decomposition of lubricants through catalytic reactions and/or tribochemical reactions of water at these nascent sites, the amount of hydrogen diffusion being proportional to wear on the steel [9, 10].

It is important to note that the FAG-FE8 set-up does not use any additional external contributors that can influence hydrogen concentration, i.e. hydrogen charging, adding water, and thus it is proposed that the lubricant is the source of hydrogen. However, it is unknown how much water may be in the oil, where small amounts may be sufficient to create hydrogen, and thus hydrocarbons may not be the only source of hydrogen in the oil. One argument for diffusible hydrogen being unnecessary in the formation of WECs is through a study conducted using fluorinated oil (free of hydrogen molecules) where WECs were created [56]; however, again water contents were not measured, and thus it is unknown whether small amounts in the oil could have generated sufficient amounts of hydrogen to drive WEC growth.

Studies have shown that at certain thresholds for slip energy criteria (PVmax, the product of contact pressure P and slip velocity V, MPa ms^−1^), WSF prevails, where locally higher permeation of hydrogen was found in these zones. The concept of slip energy criteria has been further developed, represented by the slip energy criteria per film thickness sheared (N.V/*λ*, *λ* = lambda ratio, *N* = normal load, N ms^−1^) and is based upon data obtained from different test rigs [57] to determine a threshold for WEC formation in most roller bearing configurations. It is postulated that this threshold could exist due to the fact that sliding energy generates local flash temperatures influencing the tribochemical reactions taking place at nascent surfaces [57]. Limitations, however, do exist, as this criterion does not take into account the lubricant formulation. For example, in this study the ‘special’ oil used is known to promote WSF.

Through serial sectioning analysis conducted by the authors, it has been shown that the localisation of WEC formations coincides with areas supported by thresholds for slip energy criteria and asperity energy friction accumulation, and these results are shown briefly in Fig. 6, where more details are available in [ref paper 1]. This suggests that the combination of slip/frictional energy input into the system and diffusion of hydrogen during operation may influence the propensity for WEC formations. Hydrogen diffusion analysis through finite element simulations attempts to link asperity friction energy with the absorption of hydrogen to explain the vulnerability of bearings to WEC formations in FE8 tested cylindrical roller thrust bearings [58]. Simulations show higher hydrogen concentrations in areas of high slip energy and asperity friction accumulation. Simulations, however, do reveal that the rollers hydrogen concentration, in all cases except for long running times under the assumption that no hydrogen flux crosses the surface, is below that of the washer. This is explained by shorter hydrogen absorption and emission location at the roller and free surface compared to the raceway. In addition, after a certain time is reached hydrogen concentrations in the roller exceed the washer due to the smaller relative volume of the rollers. The regeneration time, or time span between contact load cycles, could also be thought to effect the steel-to-steel contact duration. Regeneration times in rollers are lower than the raceway, this would in turn effect the time (‘wear time’) for oxide film to regenerate, hydrogen diffusion being inhibited by the protective passivating reaction layer at the surface where a nascent surface is needed for electrochemical desorption and chemisorption to occur [59, 60]. This conflicts with the results found in this study where negligible hydrogen concentrations are measured in the raceway relative to the rollers, and this discrepancy is, however, not fully understood given that similar test conditions and material were used. This discrepancy could be explained through steel cleanliness analysis conducted by the authors [ref paper 1], where the cleanliness of the raceway was found to be significantly ‘cleaner’ than the rollers. Therefore, this inconsistency could be due to differences in the cleanliness of the materials used between studies, where there is a lack of inclusion sites readily available to trap hydrogen. Evidence for the degree of boundary lubrication (the range of *λ*) controlling the propensity for WEC formation is also suggested, more WECs forming for more severe boundary regimes (*λ* in the range of 0.06–0.7) [61]. It could be reasoned that a more severe contact condition results in additional asperity contact and thus wear-induced nascent surface exposure for hydrogen generation and diffusion to take place.

### Thermal Desorption Analysis (TDA)

TDA has shown that hydrogen has diffused into the bearing steel rollers during RCF (see Figs. 3, 4), higher concentrations of diffusible hydrogen being measured for longer test durations. TDA using set-ups 1 and 2 has also shown that negligible amounts of hydrogen have diffused into the raceways during RCF operation, concentrations being similar to the 0 h test. TDA of outer and inner roller halves at 18 h showed no significant differences, suggesting that the effect of these two zones has not influenced the generation and diffusion of hydrogen.

For the 0-h test, negligible concentrations are expected since the tempering stage at ~ 200 °C would allow any weakly trapped hydrogen in the steel from manufacture to escape, in addition to any hydrogen that could have entered into the steel from final manufacturing processes would have desorbed out at room temperature over time.

For the rolling elements at 2–4 h, hydrogen concentrations were similar to the 0-h sample with no significant increases. This is logical, as 2–4 h is short test duration, and thus there is only a short time frame in which hydrogen can be generated and diffuse into the steel. Through contact surface inspection of rollers from the same test bearing, very little surface damage and no evidence of surface micro-cracking were also found at these early stages of RCF operation (see [ref paper 1] for further details), where surface micro-cracks and spall sites can act as sumps and zones for lubricant penetration and subsequent hydrogen generation.

At 6 h, elevated concentrations were measured when compared to 0–4 h. It is proposed that at 6 h, a significant time had been reached for sufficient tribochemical reactions at wear-induced nascent surfaces or areas of heterogeneous tribofilm to occur generating hydrogen.

TDA at 12 h showed comparable hydrogen concentrations to the 6-h test. It could be thought that at 12-h hydrogen concentrations would be higher than at 6 h; however, this may partly be driven by variability in rollers and RCF tests (see Fig. 3). It could be argued that the increased concentrations are due to lubricant penetration into surface micro-cracks. However, again through contact surface analysis of rollers from the same test bearing conducted in [ref paper 1], SEM showed no evidence of surface micro-cracks; a more comprehensive analysis, however, should be conducted. In addition, serial sectioning analysis from the same study showed a minimal amount of cracks connecting to the contact surface, and all rollers analysed between 0 and 12 h were also non-spalled. Therefore, lubricant penetration into surface micro-cracks is infeasible, and elevated hydrogen concentrations are proposed to be the result of wear-induced nascent surfaces and heterogeneous tribofilm forming.

TDA was also conducted on non-spalled and spalled rollers at 16.5 and 18 h with no significant differences being found between them. Serial sectioning analysis of rollers from the same test bearing at 18 h revealed a number of very small crack connections relative to the extent of the WEC network, see [ref paper 1] for further details. It is proposed that these surface connections would be insufficient to allow adequate lubricant penetration for hydrogen generation to occur. To investigate the nature of nascent surface and heterogeneous tribofilm formation during operation and to further develop the mechanism hypothesis, analysis is being conducted by the authors and will be presented in a future paper.

As hydrogen concentrations for 0–4 h are comparable, it is proposed that the hydrogen measured is trapped hydrogen from short RCF times (where if the amount of diffusible hydrogen is too small, it would not be measurable by TDA), manufacturing processes or minor contaminants. The increase in hydrogen concentration from 4 to 18 h is a reflection of the trend for increased hydrogen diffusion during RCF operation.

**Fig. 5:**
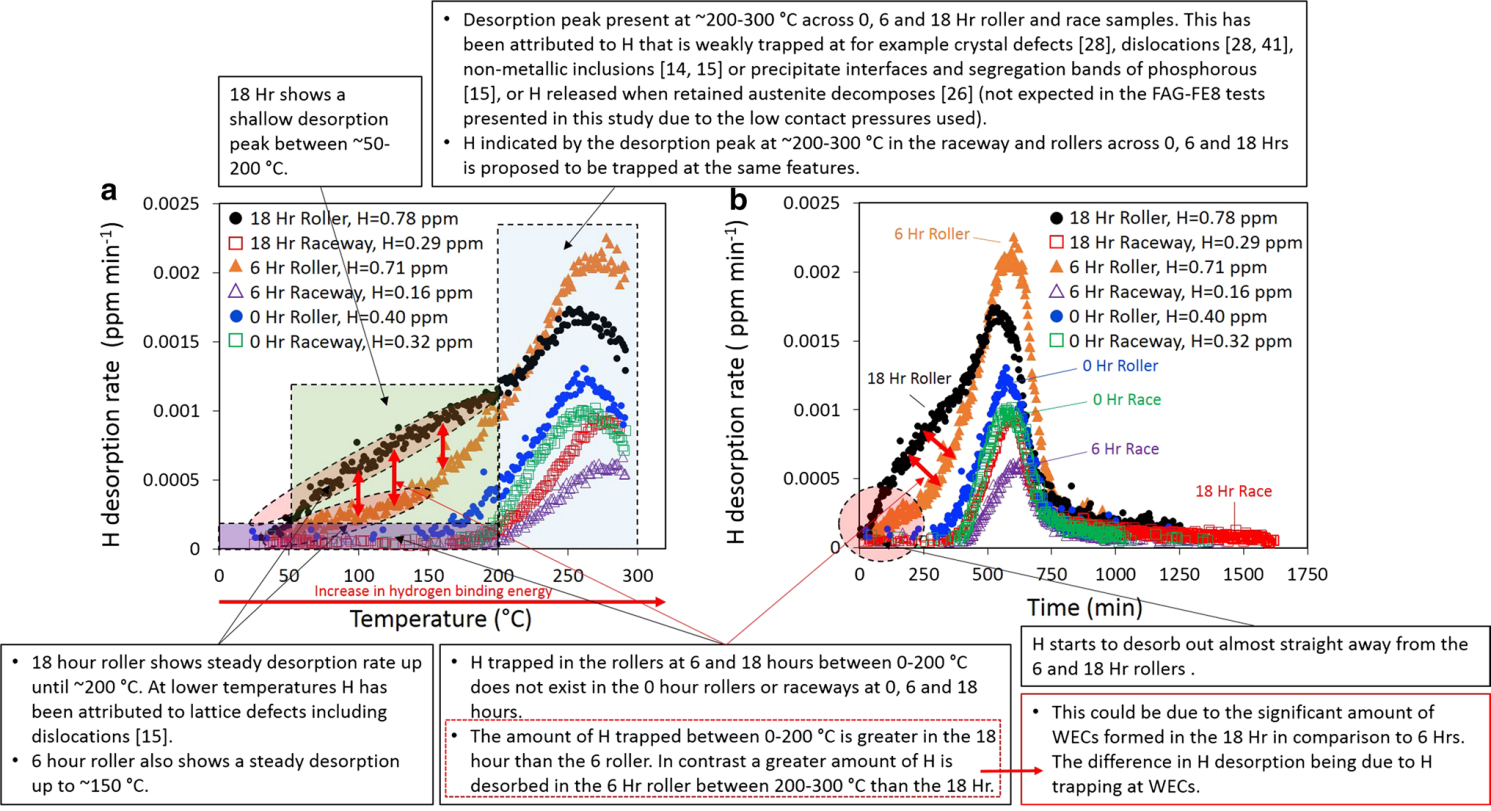
Annotated discussion points of key features from TDA set-up 2 results shown in Fig. 4

Figure 5 discusses the key features found from the results obtained by TDA set-up 2 and is detailed in Fig. 4.

During RCF, hydrogen generated at the time of operation was at that point in its diffusible state. This hydrogen can then diffuse and become attracted to the various trapping sites within the steel matrix, some of this hydrogen becoming strongly trapped. Therefore, if it is to be contested as to what is considered ‘diffusible’ or ‘non-diffusible’ hydrogen at temperatures ≤ 400 °C, then one could consider that hydrogen that has become strongly trapped or ‘non-diffusible’ (that was once ‘diffusible’ during RCF operation) is still a measure of the hydrogen that has ‘diffused’ into the steel at some point during operation. It is also important to note that at higher TDA temperatures (1100 °C) conducted post-1st round TDA at ≤ 400 °C on the 16.5 h rollers (see Table 2), additional hydrogen was measured (0.30 ppm). This is classed as ‘non-diffusible’ strongly/residually trapped hydrogen (sourced during operation or from manufacturing processes). Therefore, not all (or any) of the ‘non-diffusible’ strongly trapped/residual hydrogen was released when conducting TDA at ≤ 400 °C and so the hydrogen measured is considered to be weakly trapped ‘diffusible’ hydrogen.

It can be seen in one case (6 h rollers) that an increased concentration of diffusible hydrogen was measured when analysing 2× rollers simultaneously as opposed to a single rolling element (0.18–0.37 ppm). This could be due to a lack of total hydrogen evolved by the single roller to be detectable by the TDA, variability between different rolling elements, an increase in the surface area analysed, increased chance of surface contaminants and residue and oxide layer, though strict measures were taken to reduce the possibility of this effect.

Raceway washers and rollers in some cases were cut into relevant sections before TDA. The heat generated due to cutting would lead to hydrogen losses; however, as it can be seen from the TDA results, no significant differences in hydrogen concentrations were measured when analysing rolling element halves and whole rolling elements and so the cutting has not significantly influenced the TDA measurement.

The values measured by TDA between the two test set-ups are comparable. The results measured by test set-up 2 are higher than those measured using set-up 1 by ~ 0.20–0.30 ppm; however, this can be regarded as an offset between set-ups. Therefore, the trend for elevated hydrogen concentrations being measured over increasing test duration in the rollers and negligible concentrations measured in the raceways still holds. In terms of absolute bulk concentrations, these results are not readily comparable between other studies that have used different preparation techniques and test machines due to uncertainties and experimental factors when performing hydrogen measurements. They are, however, comparable between the multiple trips and analyses conducted by the authors using these set-ups. Note also that these bulk values reported are not representative of concentrations found within certain zones of the steel (e.g. near surface), nor locally where hydrogen is attracted at traps such as crack tips [28, 60], areas of plastic deformation and inclusions [14]. It is likely the concentration at these locations will be higher due to hydrogen being attracted and trapped at these sites [28, 29, 60].

In summary, the major findings are as follows,

- In comparison with the baseline 0-h hydrogen concentration measurement, for longer test durations the trend for increased hydrogen concentration measured in the rollers has been shown through TDA. Therefore, TDA on two independent instrument set-ups has revealed that hydrogen *has* diffused into the bearing rollers during RCF operation, higher concentrations being measured for longer test durations.
- TDA has shown negligible amounts of hydrogen has diffused into the raceways during RCF operation. The authors are unable to confirm the weight of each factor at this point; however, it is likely that the difference in dynamics experienced on the raceway and how this affects the tribofilm and wear are responsible. In addition, factors including hardness difference between the raceway and roller and steel cleanliness could also be influential.
- It is the authors’ opinion that the hydrogen measured ≤ 400 °C is a valid measure of the mobile ‘diffusible’ hydrogen, hydrogen measured above this being ‘non-diffusible’. It is considered that during RCF operation, strongly trapped ‘non-diffusible’ hydrogen is still a measure of hydrogen that was at one point during operation ‘diffusible’. Additional hydrogen was also measured at higher temperatures (1100 °C) post-TDA at ≤ 400 °C on selected samples.
- TDA using set-up 2 has revealed a number of factors in regard to the diffusion of hydrogen during RCF operation. The authors cannot explicitly explain each factor through the results obtained in this study; however, it is considered that the introduction of WECs affects the trapping of hydrogen.

### TDA Relation to WEC Formations

Metallographic analysis has been conducted on rollers from the same bearing under the same test conditions used in this study to record and map WECs through serial sectioning, comprehensive details being described in [ref paper 1]. The following section combines these results along with the TDA conducted in this study to discuss the relationships between WEC formations and diffusion of hydrogen.

Progressive wear, very little surface damage and no cracks making contact surface connections were observed between 2 and 12 h, with only a small number of very small/short surface crack volume connections being found at 18 h [ref paper 1]. It is reasoned that such small/short interactions recorded at 18 h would not drive extensive WEC networks in the subsurface or allow sufficient lubricant penetration into the crack to aid in WEC growth. Therefore, through the combined TDA and metallographic analysis, evidence shows that the mechanism of hydrogen entry is diffusion through wear-induced nascent surfaces or areas of heterogeneous tribofilm, hydrogen being generated through the decomposition of lubricant through catalytic reactions [9, 10]. However, as discussed in Sect. 4.1, it should be noted that small amounts of water in the oil can generate sufficient amounts of hydrogen. Therefore, without measuring the water content in the oil it is unknown whether small amounts of water are present and that significant amounts of hydrogen were also generated through tribochemical reactions with water [3]. It is, however, also reported that when water is present, hydrogen predominantly derives from the oil opposed to the water [62]. Analysis is currently being conducted by the authors to understand chemical/tribofilm heterogeneity effects and mechanisms.

**Fig. 6:**
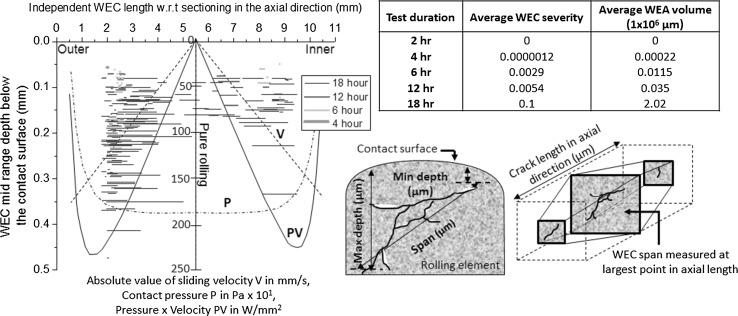
Distribution of the individual WECs recorded in 4–18-h rollers through metallographic analysis conducted in. The table shows the average WEC severity and WEA volume recorded for each test duration. Schematic illustrates relevant dimensions recorded for individual WECs. Adapted from [ref paper 1]

**Fig. 7:**
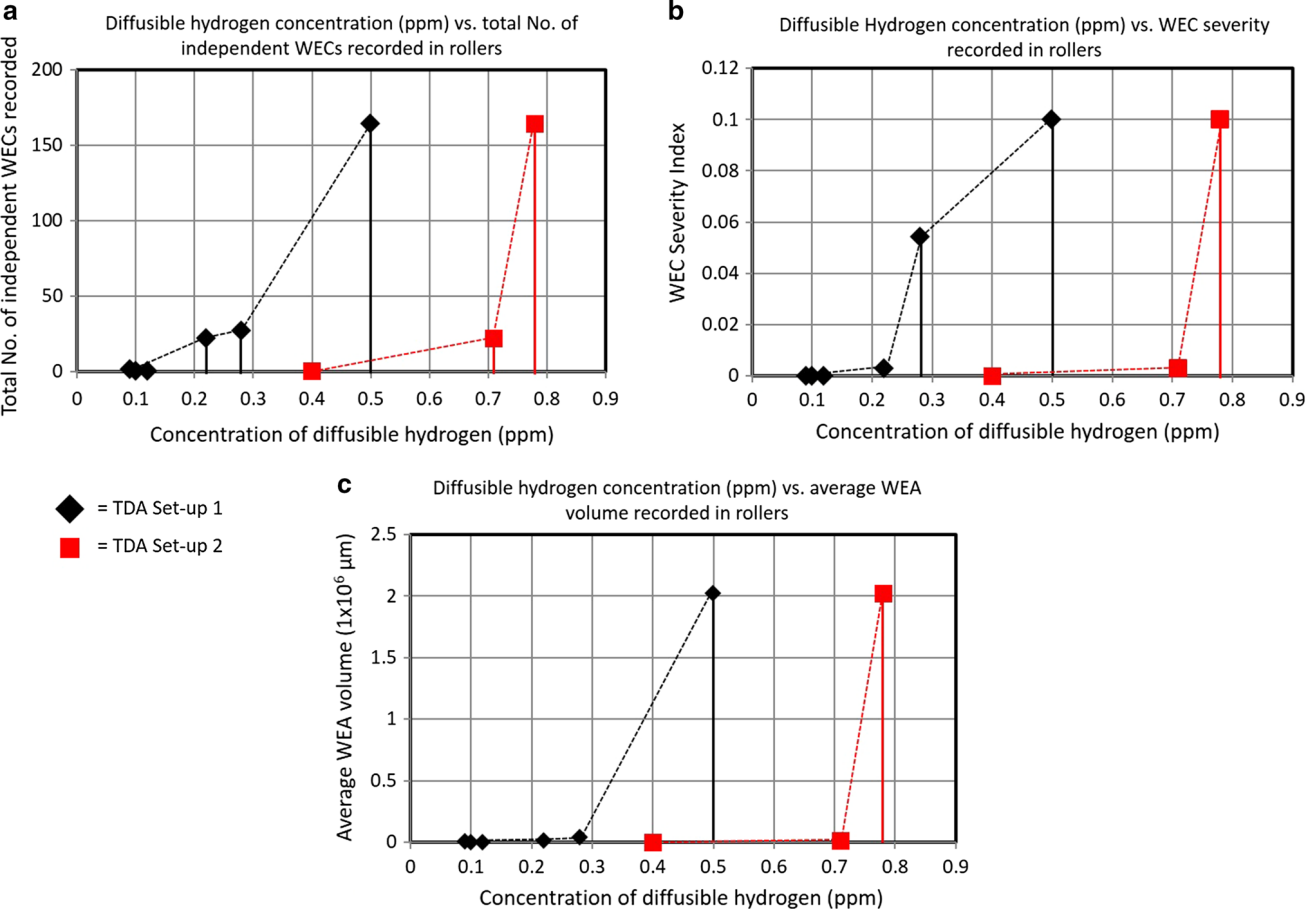
Relationship between the concentration of diffusible hydrogen measured for set-ups 1 and 2 and WEC formations recorded through metallographic analysis in [ref paper 1]. **a** Diffusible hydrogen concentration versus the total number of independent WECs recorded in the rolling elements. **b** Diffusible hydrogen concentration versus average severity for WEC formations in the rolling elements. **c** Diffusible hydrogen concentration versus average WEA volume recorded in the rolling elements

It has been shown by the authors [ref paper 1] that the propensity and average size of WEC formations increase with test duration from 0 to 18 h, a ramped increase being seen at the later stages of RCF operation (12–18 h), and this result is shown in Fig. 6. Figure 7a shows the relationship between measured diffusible hydrogen concentration for set-ups 1 and 2 and the total number of independent WECs recorded from 0 to 18 h during metallographic analysis that are displayed in Fig. 6. In addition, the relationship between diffusible hydrogen concentration and severity index for WEC formation (Fig. 7b) and the diffusible hydrogen concentration versus average WEA volume measured (Fig. 7c) are also shown. The WEC severity index is calculated and weighted upon the combined WEC length in the axial roller direction, WEC radial size (maximum depth minus the minimum depth the WEC propagates into the subsurface) and WEC span, see Fig. 6. The average WEA volume is a measure of the total amount of WEA found in association with a crack, this being calculated through examination of a number of WECs for each test duration and individually measuring the total WEA found at varying slice intervals through a whole 3D WEC network. The average WEC severity and WEA volume for each test duration are shown in Fig. 6, further details of which can be found in [ref paper 1]. The combined results in Fig. 7 indicate that a link exists between diffusible hydrogen concentration and the formation of WECs. An increase in WEC formations accompanying elevated hydrogen concentrations. Through the combined results discussed above, it is proposed that hydrogen that has diffused into the steel matrix during RCF operation can aid in the formation and propagation of WECs in the subsurface.

Evidence for WECs recorded at short RCF test times has been found through serial sectioning analysis of 4-h rollers [ref paper 1] (see Fig. 6). TDA for the 4-h rollers measured negligible concentrations of diffusible hydrogen, comparable to the 0 and 2-h tests (see Fig. 3). This, however, may have been due to the lack of a sufficient volume of hydrogen being effused for the detection limit of the TDA analyser. Considering the case if there was indeed no increase in hydrogen concentration at the 4-h roller. Since evidence for WEC initiation was consequently found at 4 h, either (1) the local concentration of hydrogen was relatively high at the inclusion or crack to aid in crack initiation as opposed to the bulk average of the steel, or (2) hydrogen has not aided in the initiation stage of this WEC, rather hydrogen accelerated crack propagation, this only occurring once additional hydrogen has diffused into the steel above some threshold. This is supported as for longer RCF test durations (6–18 h) an increase in WEC formation and size accompanies an increase in diffusible hydrogen concentration. It may be that a sufficient threshold concentration of diffusible hydrogen is reached accelerating WEC formations where hydrogen acts to decrease the Mode I/II stress limits for crack growth and propagation [40, 63]. Hydrogen acceleration of WECs is also supported by the fact that under the test conditions used in this study (*P*_max_ 1.5–1.9 GPa (depending on contact length used between 7 and 9 mm) and low RCF test times) flaking is not known to normally occur with other lubricants.

It has been shown through serial sectioning [ref paper 1] that the total WEA volume found associated with the crack increases with RCF test duration with a step increase in the total WEA volume being recorded at the later stages of RCF operation between 12 and 18 h (see Fig. 6). From Fig. 7c, it can be seen that the average WEA volume increases for an increase in hydrogen concentration, a significant step increase occurring for hydrogen concentrations beyond ~ 0.3 ppm. Therefore, it is hypothesised that the diffusion of hydrogen can aid in the formation of WEAs. This appears to be at a greater extent at the later stages of RCF duration, the diffusion of hydrogen promoting WEC growth. Supporting evidence for the crack being a prerequisite to the WEA is shown in [ref paper 1], a possible mechanism for this being crack face rubbing/beating [28, 29], it is thus proposed that the addition of hydrogen aids in crack growth thus enhancing the ability for crack face rubbing/beating to occur in the formation of WEAs.

Metallographic analysis has also revealed that a large number of small near surface (< 25 μm) WEA/WECs formed in 18-h rollers, examples of which can be seen in [ref paper 1]. One influencing factor in the formation of these features is considered to be due to the increased diffusible hydrogen concentration between 12 and 18 h, where locally at the near surface region, higher localised penetration and concentrations of diffusible hydrogen may exist than in the depth of the steel.

It has also been revealed that the outer and inner roller halve zones significantly influence the propensity for WECs to form, the outer half being considerably more dominant in the formation of WECs [ref paper 1], see Fig. 6. However, TDA of outer and inner roller halves showed no significant differences in measured hydrogen concentration; therefore, it was not a difference in concentration that contributed to the WEC formations. Studies have shown increased hydrogen permeation in zones of high PVmax (slip energy criteria) [51, 64, 65], these zones coinciding with WSF where proposed thresholds for slip energy criteria correspond to a ~ 2-mm zone from either the outer or inner roller edge (see Fig. 6) for the FAG-FE8 set-up [57]. Metallographic analysis [ref paper 1] has revealed that a higher density of WEC formations is found in this ~ 2 mm zone (see Fig. 6); therefore, analysis to measure the concentration of hydrogen in this specific zone may be more applicable.

TDA of raceway washer sections revealed on average lower concentrations of diffusible hydrogen than those measured in the rollers, see Table 2. Metallographic analysis of raceway washer sections at 18 h [ref paper 1] and at 18.5 h [41] showed no signs of ‘conventional’ WEC/WEAs, this being in contrast to the extensive number of WECs found in the rollers. It is proposed that one possible reason as to why no WECs were found in the raceways is due to the lack of diffusible hydrogen available to accelerate crack growth. The raceway is also ~ 23% softer (590 HV) than the rollers (765 HV); therefore, the raceway is less prone to cracking due to an increased toughness. It is well recognised that hydrogen effects higher strength steels, hydrogen embrittlement occurring when hydrogen is in its atomic mobile form, hydrogen able to retain its mobility in high strength steels [11]. Hydrogen has, however, been shown to have little influence on toughness and no effect on the hardness of 100Cr6 bearings steel [50]. There are a number of theories that try to explain the effect of hydrogen on high strength bearing steels (see Sect. 1); however, no agreement yet has been made. Steel cleanliness analysis has shown that the raceway is significantly cleaner than the rollers [ref paper 1]. The reduced cleanliness and thus density of inclusions indicate that there are less available inclusion sites for hydrogen to become trapped, this perhaps providing supporting evidence for the lack of diffusible hydrogen measured in the raceways. Differences in solubility between steels may also have an effect on the diffusion of hydrogen. While the solubility has not been checked by the authors, both roller and raceway are the same steel type from the same bearing, the only difference being hardness, and therefore solubility differences are suggested to be negligible. At this point, the authors are unable to confirm the significance of each of these factors on influencing hydrogen permeation. However, it is likely that a combination of these factors, alongside differences in the dynamics experienced by the raceway and how this affects the tribofilm and wear could be the answer.

### Influence of Oil

A number of additives found in lubricants have been shown to promote WSF occurrence, these include; extreme pressure (EP) and anti-wear (AW) additives consisting of sulphur and phosphorus compounds [66, 67], where sulphur aids in hydrogen diffusion by preventing atomic hydrogen recombination [68] and formulations of AW zinc dithiophosphates (ZDDP/ZnDTP/ZnDDP) with detergent/rust preventative calcium sulphonate additives [6, 61, 69–74].

A link between oil additives and the formation of WSF/WECs clearly exists; however, further investigation is needed to understand the effects and relationships of the additives. The ‘special’ oil used in this study has shown that a relationship exists between the propensity for WEC formations and the diffusion of hydrogen into the bearing steel during RCF operation. This ‘special’ oil contains mixes of calcium sulphonate and ZDDP additives (as well as other potentially influential additives such as sodium sulphonates, EP additives and friction modifiers); therefore, the relationship between calcium sulphonate and ZDDP mixes, diffusion of hydrogen and the propensity for WEC formations is currently being investigated and will be presented in a future study by the authors.

## Conclusions

TDA through two set-ups has been used to measure the concentration of diffusible hydrogen in standard 100Cr6 bearing steel. This has been conducted for the first time under non-hydrogen-charged conditions and over increasing RCF test durations, the aim being to investigate the mechanism of hydrogen diffusion and the relationship between measured diffusible hydrogen concentration and operation time. In addition, the link between hydrogen diffusion and the propensity for WEC formations recorded through metallographic analysis conducted in [ref paper 1] has been explored.

1. TDA on two independent set-up instruments revealed that hydrogen diffused into the rolling elements during RCF operation, higher concentrations being measured for longer test durations. Negligible concentrations were measured in the raceway washers over all test durations.
2. There is debate as to what state hydrogen is when measured at ≤ 400 °C, whether it is mobile ‘diffusible’ or whether an amount of non-mobile, ‘non-diffusible’ hydrogen is also measured. TDA using two experimental set-ups has shown correlation between results, where it is of the author’s opinion that the state of hydrogen recorded at ≤ 400 °C is an applicable measure of the mobile ‘diffusible’ hydrogen that has entered the steel during RCF operation. Above this temperature, it is considered ‘non-diffusible’.
3. TDA coupled with extensive metallographic analysis conducted in a separate study by the author’s has shown that;
  A relationship exists between the diffusion of hydrogen and the propensity for WEC formations, this also correlating directly with the volume of WEA and WEC severity index.
  Metallographic analysis has shown no evidence for conventional WEA/WECs in the raceway washers, it is suggested that this may be due to the lack of hydrogen readily available to accelerate WEC formations and the raceways higher toughness retarding crack initiation/propagation. Steel cleanliness analysis has also shown the raceway washer to be far ‘cleaner’ than the rollers, and thus less crack initiation sites may have been available for WECs. It is also suggested that an absence of WEC formations and negligible hydrogen concentrations measured may be due to the lack of inclusion sites available to trap hydrogen.
  Hydrogen diffusion into the steel during operation is thus proposed to act as an accelerator in WEC formations and propagation in the subsurface. The mechanism of hydrogen entry into the steel is suggested to be diffusion at wear-induced nascent surface or areas of heterogeneous/patchy tribofilm, since most WECs did not breach the contact surface, and those that did only had very small crack volumes for lubricant entry to occur. Hydrogen would be generated by decomposition of lubricant through catalytic reactions and/or tribochemical reactions of water, where measurements for small amounts of water in the oil should be taken.

## Appendix

The minimum oil film thickness (*h*_min_) between the rolling elements and raceway washer was calculated using Hamrock–Dowson visco-elastic calculation [53, 54], see Eq. (1).

1 $$ h_{ \hbox{min} } = 3.63{\text{U}}0^{.68} {\text{G}}^{0.49} {\text{W}}^{ - 0.073} \left( {1 - {\text{e}}^{ - 0.68k} } \right) $$

where *h*_min_ minimum film thickness (m); *U* dimensionless speed parameter, *uη*_0_/(*E*′*R*); *G* dimensionless material parameter, *αE*′*; W* dimensionless load parameter, *N*/(*E*′*R* ^2^); *u* mean lubricant entrainment speed (m/s); *η*_0_ viscosity at atmospheric pressure of lubricant (Pa s); *E*′ effective elastic modulus, [75]; *R* reduced radius of curvature (m); *α* pressure–viscosity coefficient (Pa^−1^); *N* normal load (N); *k* ellipticity parameter defined as *k* = *a*/*b*, where ‘*a*’ is the semiaxis of the contact ellipse in the transverse direction (m) and ‘*b*’ is the semiaxis in the direction of motion (m). For line contact, *k* = ∞.The initial lambda ratio (*λ*) was then calculated using Eq. (2), where Rq,1 and Rq,2 are the rms roughness values of the two contact surfaces.

2 $$ \lambda = h_{\hbox{min} } /\left( {Rq,1^{2} + \, Rq,2^{2} } \right)^{1/2} $$
